# The Impact of Point-of-Care Testing for Influenza on Antimicrobial Stewardship in UK Primary Care: Nested Cohort Study

**DOI:** 10.2196/72322

**Published:** 2025-07-15

**Authors:** Uy Hoang, Jessica Smylie, Elizabeth Button, Jack Macartney, Cecilia Okusi, Rachel Byford, Filipa Ferreira, Charis Xie, Mark Joy, Tristan Clark, Simon de Lusignan

**Affiliations:** 1 Nuffield Department of Primary Care Health Sciences, Medical Sciences Division, University of Oxford, Gibson Building, Radcliffe Observatory Quarter, Woodstock Road, Oxford, OX2 6GG, United Kingdom, 44 1865289344; 2 Barts and The London School of Medicine and Dentistry, Wolfson Institute of Population Health, Queen Mary University of London, London, United Kingdom; 3 Faculty of Medicine, School of Clinical and Experimental Sciences, University of Southampton, Southampton, United Kingdom; 4 Royal College of General Practitioners, London, United Kingdom

**Keywords:** medical records systems, computerized, influenza, point of care systems, primary care, implementation, outcome assessment, health care

## Abstract

**Background:**

Influenza and respiratory syncytial virus (RSV) predominantly circulate during the winter season and cause acute respiratory illness (ARI). Deploying molecular point-of-care testing (POCT) in primary care can inform whether a patient presenting with an ARI has influenza or RSV. An early virological diagnosis could facilitate appropriate use of antivirals and enable better antimicrobial stewardship.

**Objective:**

This study aimed to report the impact of POCT for influenza and RSV on antimicrobial prescribing, including antiviral therapy in primary care.

**Methods:**

The impact of POCT for influenza on antimicrobial stewardship (PIAMS) in UK primary care was a nested cohort study undertaken from January 20 to May 31, 2023, after the period of peak virus circulation, within practices that contribute data to the English sentinel network. People presenting with ARI had a nasopharyngeal swab performed and were tested for influenza and RSV with a molecular POCT analyzer located within the practice. Data on antimicrobial prescribing and other study outcomes were collected by linking information from the analyzer to coded data from the patient’s computerized medical record.

**Results:**

In total, 323 swabs were collected from 10 PIAMS study practices. In total, 59.7% (197/323) of swabbed patients were female, and the mean age was 37.28 (SD 25.05) years. Furthermore, 2.9% (9/323) of all swabs were positive, with 0.3% (1/323) positive for influenza A, 1.6% (5/323) positive for influenza B, and 0.9% (3/323) positive for RSV. In total, 80 patients were prescribed antibiotics 7 days following POCT testing. There were no instances of antiviral prescribing in the 7 days post testing. A statistically significant difference in antibiotic prescribing given a positive POCT result compared with a negative test was not found with an unadjusted odds ratio (OR) of 7 days post testing. A statistically significant difference in antibiotic prescribing given a positive POCT result compared with a negative test was not found with an unadjusted OR of 1.54 (95% CI 0.38‐6.30; *P*=.55) and adjusted OR of 1.21 (95% CI 0.00‐1.78).

**Conclusions:**

This study illustrates the risk of having a narrow study window; our observation period was not aligned with when influenza was circulating. The peak of weekly incidence of influenza in the sentinel network was in the last week of 2022, and RSV was circulating before this. Further evidence is needed to assess the impact of POCT on antimicrobial prescribing. The viruses tested for using POCT could be aligned with the circulating viruses identified by the sentinel network.

## Introduction

Accurate, rapid molecular point-of-care testing (POCT) has the potential to (1) improve clinical decision-making regarding the use of antibiotics and antivirals, (2) improve patient outcomes due to the early appropriate use of antivirals, and (3) provide better information to inform sentinel surveillance and clinical research including studies of vaccine effectiveness and real-world trials [1,2].

For patients with influenza infection, early diagnosis and administration of antivirals may improve clinical outcomes [3,4]. They may also limit symptom duration and spread to household contacts, and newer antivirals for influenza and respiratory syncytial virus (RSV), such as Baloxavir, have been shown to improve the time to resolution of symptoms and reduce complications in high-risk patients [5].

Currently, only a small proportion of patients with acute respiratory illness (ARI) undergo diagnostic microbiological testing before receiving treatments in primary care [6], and there is evidence of widespread variations in antimicrobial prescribing practices [7]. This is important as prescribing in primary care accounts for about 8% of National Health Service expenditure, which is equivalent to over £9 billion (US $12.25 billion) per year, with just over £220 million (US $299.53 million) being spent on antimicrobials [8]. Inappropriate prescribing of antimicrobials and unwarranted variation in prescribing can contribute to an increase in antimicrobial-resistant strains and patient adverse events in the short and long term [9].

We have previously shown that in a prepandemic context, it is feasible to undertake POCT for influenza in primary care in England, with promising impacts on antimicrobial use and comparable estimates of influenza vaccine effectiveness to published data [10-12]. Although its impact on more severe outcomes, such as hospitalization and mortality, following infection was not reported.

With the ending of widespread national testing for SARS-CoV-2 and other respiratory tract infections (RTIs) in the United Kingdom in March 2022 [13], and with high levels of circulating influenza in a post–COVID-19 health service during autumn in 2022 compared with 2021, there was a need to revisit questions about the feasibility of implementing rapid diagnosis of influenza during the expected peak of viral circulation from October 2022 to May 2023 and its impact on clinical management in terms of improved antimicrobial stewardship.

We aimed to deploy POCT during October 2022 to May 2023. The impact of POCT for influenza on antimicrobial stewardship (PIAMS) took place between January 20, 2023, and May 31, 2023, after the peak of virus circulation.

## Methods

### Study Design, Setting, and Population

This cohort study was nested within the English National Sentinel Surveillance Network managed by the Oxford Royal College of General Practitioners (RCGP) Research and Surveillance Centre (RSC).

The RSC network of over 2000 primary care practices in England is generally representative of the English population [14] and serves as the English national infectious disease surveillance network. It has been providing weekly data extracts for over 50 years, which are used to monitor trends in infectious disease and investigate real-world vaccine and treatment effectiveness [15]. A subset of practices within the network undertake virology swabbing for testing at the UK Health Security Agency’s reference laboratory [14].

**Figure 1.**
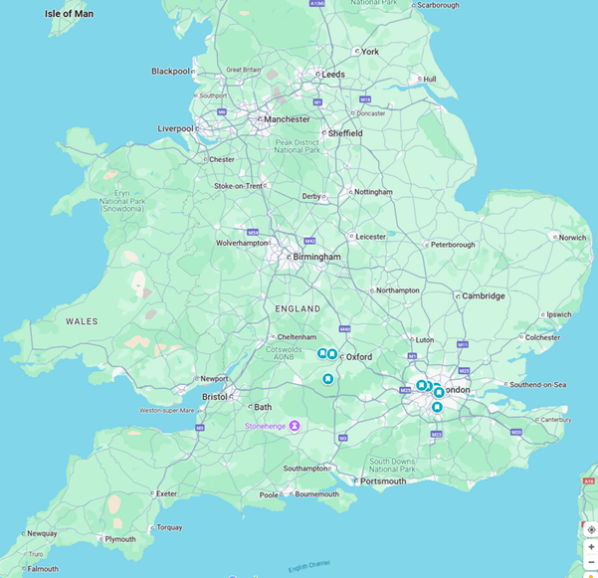
Location of general practices in the point-of-care testing influenza on antimicrobial stewardship (PIAMS) study, a nested cohort study undertaken within the English National Sentinel Surveillance Network between January 20, 2023 (International Organization for Standardization week 3, 2023), and May 31, 2023 (International Organization for Standardization week 22, 2023).

All practices that contribute data to the English National Sentinel Network were invited to participate in the PIAMS study. In total, 10 practices were selected (Figure 1). We prioritized practices within the network with the capacity to undertake point-of-care influenza testing and who had previously been involved in SARS-CoV-2 POCT through the Rapid Community Testing for COVID-19 study [16]. Those practices that had a history of less than 80% complete data returns during the previous winter season were excluded. The sample size was influenced by an earlier nested cohort study of respiratory POCT undertaken before the pandemic in the United Kingdom, which used 12 primary care practices [10,17].

Each participant received training about the study, including hands-on training on how to administer a swab test and how to use the POCT analyzers.

### Case Definition of Eligible Patients

All patients registered within the PIAMS study practice and showing symptoms of influenza-like illness (ILI), ARI, or fever of higher than 37.5 °C were eligible for the study if they consented to participate. We used the European Centre for Disease Prevention and Control case definitions of ILI and ARI for this study. We used the following exclusion criteria: (1) the patient has an opt-out code on their medical record, and (2) the patient declined informed consent.

### Face-to-Face Recruitment of Eligible Patients and POCT for Influenza

We undertook opportunistic swab sampling for this study, with potential participants being identified from those registered patients who presented to the PIAMS study practices with respiratory symptoms described in the case definition. No screening or eligibility assessment was undertaken. Blinding of participants and researchers was not undertaken.

Eligible patients or their parents or legal guardians were approached by a practice general practitioner (GP) or research nurse to explain the study and ask for consent to take part when they presented for a face-to-face consultation at the practice.

After obtaining consent, a nasopharyngeal swab was taken by a suitably qualified and experienced GP or research nurse. For those who do not attend the practice in person, a self-test kit was sent to their home.

The swab was inoculated in a test kit and tested with the POCT analyzer as soon as possible after being taken. The results were available to the clinician in less than 20 minutes.

The POCT test we used was the cobas liat analyzer, manufactured by Roche Diagnostics International [18]. This POCT analyzer is an automated multiplex polymerase chain reaction system, with previous studies demonstrating excellent performance comparable with gold standard laboratory assays, with sensitivity/specificity in the region of 100%/97.1% for influenza A, 97.8%/99.7% for influenza B, and 94.2%/99% for RSV when fresh prospectively collected samples are tested [19,20]. It has Conformité européenne (CE) marking in the European Union [21]. In the United States, it is approved by the Federal Drug Administration [22].

All eligible patients for this study were seen face-to-face.

### Study Outcomes and Data Sources

Antimicrobial prescribing and other study outcomes for those who had been swabbed were obtained by linking information from the POCT with data from the patient’s computerized medical record in primary care. A pseudonymized National Health Service number was used to allow the linkage of these datasets and to ensure patients’ records were kept confidential.

Data for the study are held on dedicated secure servers within the Oxford-RCGP Clinical Informatics Digital Hub trusted research environment. The research group’s secure network is situated behind a firewall within the university’s network. To protect privacy and confidentiality, only study staff or associated members of the research group who have been appropriately trained and approved by the Head of Department can access the data from secure workstations or secure laptops with encrypted drives. All staff members of the research group working within the team must work from secure workstations or secure laptops with encrypted drives within the research group’s secure network. A risk assessment of the physical security of the research group’s offices and server room has been conducted by the building and facilities manager, the faculty information technology service manager, and the research group’s information governance lead. The university is compliant with the Data Protection Act and UK General Data Protection Regulation and has systems for technical and organizational controls for information security, including a university-level information security and governance group, chaired by the university senior information risk owner. The research group’s private network has its own system-level security policy and is tested for vulnerabilities annually.

### Statistical Analysis

To quantify the impact of POCT for influenza on antimicrobial prescribing in primary care, we present the odds ratio (OR) of antimicrobial prescribing given a positive POCT result compared with a negative POCT test. Unadjusted OR was calculated by dividing the odds of antimicrobial prescribing in those with a positive POCT result group by the odds of antimicrobial prescribing in those with a negative POCT result. We used established methods to calculate the standard error and 95% CI for the OR [23], as well as the *P* value for significance [24]. The unadjusted OR for antibiotic prescribing and antiviral prescribing was calculated separately. We also used logistic regression to calculate adjusted ORs taking into account patient demographics (age, sex, ethnicity, and socioeconomic status as measured using the Index of Multiple Deprivation), urban-rural classification, and smoking status, factors known to be associated with antimicrobial prescribing in patients presenting with respiratory illness [25]. Missing values were excluded.

### Ethical Considerations

The study was reviewed and approved by the English National Research Ethics Committee (reference 21/YH/0077) and Integrated Research Application System (reference 292961), dated October 5, 2022.

Study practices were given a stipend to cover the costs of training staff members and hosting the study. A small remuneration was also provided to practices for each POCT swab to cover the additional time taken during each consultation to undertake swabbing for this study. Patients were not remunerated for taking part in this study. Informed consent was undertaken by a trained practice GP or research nurse for all patients who took part in this study. A pseudonymized extract of information from consented patients was analyzed for this study.

## Results

### PIAMS Practice Recruitment

In total, 10 practices were recruited for the PIAMS study with a total registered population size of 144,426. The demographic profile of the PIAMS practices is illustrated in Table 1.

**Table 1.** Characteristics of study practices taking part in the point-of-care testing influenza on antimicrobial stewardship (PIAMS) study, a nested cohort study undertaken within the English National Sentinel Surveillance Network between January 20, 2023 (International Organization for Standardization week 3, 2023), and May 31, 2023 (International Organization for Standardization week 22, 2023).

Practice or characteristics	1	2	3	4	5	6	7	8	9	10	England or Wales
Practice size^a^, n	18,128	18,324	8909	7305	9434	16,435	15,672	10,541	19,125	20,553	59,620,100
Female^a^, n (%)	9344 (51.5)	8056 (44.0)	4354 (48.9)	3560 (48.7)	4606 (48.8)	8142 (49.5)	7961 (50.8)	5090 (48.3)	9099 (47.6)	10,483 (51.0)	30,420,202(51.0)
Ethnicity, n/N (%)^b^											
White	8669/8953 (96.8)	1628/13,588 (12.0)	1917/3196 (60.0)	3748/7612 (49.2)	3748/7612 (49.2)	5844/10,678 (54.7)	5196/5398 (96.2)	4566/8483 (53.8)	5745/18,754 (30.6)	4622/4870 (94.9)	48,209,395/56,075,912 (86.0)
Mixed or multiple ethnic groups	112/8953 (1.3)	347/13,588 (2.6)	156/3196 (4.9)	408/7612 (5.4)	408/7612 (5.4)	624/10,678 (5.8)	83/5398 (1.5)	568/8483 (6.7)	6.7 (1250/ 18,754 (6.7)	77/4870 (1.6)	1,224,400/56,075,912 (2.2)
Asian or Asian British	132/8953 (1.5)	10,041/13,588 (73.9)	778/3196 (24.3)	2089/7612 (27.4)	2089/7612 (27.4)	1487/10,678 (13.9)	88/5398 (1.6)	1421/8483 (16.8)	6534/18,754 (34.8)	110/4870 (2.3)	4,213,531/56,075,912 (7.5)
Black, African, Caribbean, or Black British	31/8953 (0.3)	1285/13,588 (9.5)	249/3196 (7.8)	1073/7612 (14.1)	1073/7612 (14.1)	2365/10,678 (22.1)	14/5398 (0.3)	1454/8483 (17.1)	4760/18,754 (25.4)	43/4870 (0.9)	1,864,890/56,075,912 (3.3)
Other ethnic group	9/8953 (0.1)	287/13,588 (2.1)	96/3196 (3.0)	294/7612 (3.9)	294/7612 (3.9)	358/10,678 (3.4)	20/5398 (0.4)	474/8483 (5.6)	465/18,754 (2.5)	18/4870 (0.4)	563,696/56,075,912 (1.0)
IMD^c^ Decile^d^, n	10	4	3	2	2	2	10	1	5	8	—^e^

a Registered practice population as of September 2023.

b Ethnic group statistics from the 2011 Census for England and Wales at postcode sector level.

c IMD: Index of Multiple Deprivations.

d IMD decile: from 1=the most deprived 10% geographic area to 10=the least deprived 10% of geographic area.

e Not applicable.

### Swabbing Rates in PIAMS Practices

The study started on January 20, 2023 (International Organization for Standardization [ISO] week 3, 2023). In total, 323 swabs were collected from PIAMS study practices until May 31, 2023 (ISO week 22, 2023). Multimedia Appendix 1 illustrates the number of POCT swabs collected in the PIAMS study by week.

Swabbing rates varied considerably between PIAMS study practices from 4.9 to 75.2 swabs per 1000 patients with eligible symptoms (Multimedia Appendix 2).

**Figure 2.**
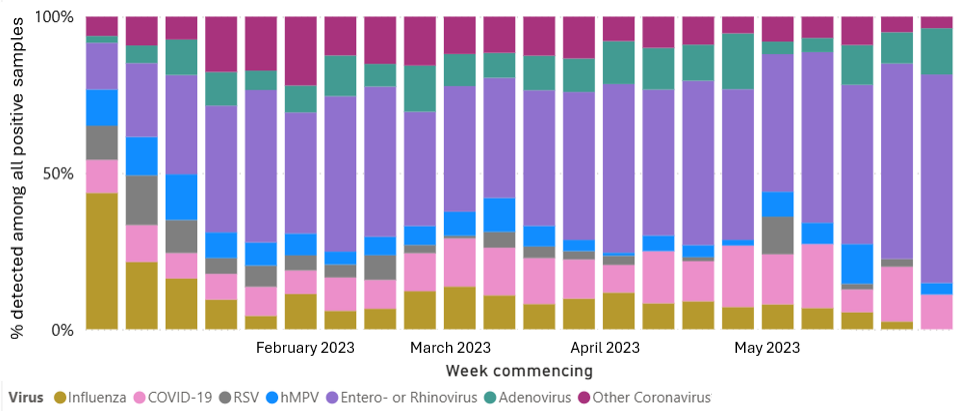
English national sentinel virology surveillance swab for respiratory syncytial virus (RSV), influenza, SARS CoV-2, and other respiratory viruses between 2022 and 2023 [26]. hMPV: human metapneumovirus.

### Summary Demographics of All Swabbed Patients

Table 2 illustrates further detailed analysis of the demographics of those swabbed, showing that 59.4% (192/323) were female, and the mean age of those swabbed was 37 years. A total of 46.4% (150/323) samples were taken from patients of White ethnicity and 39% (126/323) samples taken from patients of Asian, Black, or mixed ethnicity.

**Table 2.** Demographic profile of the patients swabbed in the point-of-care testing influenza on antimicrobial stewardship (PIAMS) study, a nested cohort study undertaken within the English National Sentinel Surveillance Network between January 20, 2023 (International Organization for Standardization week 3, 2023), and May 31, 2023 (International Organization for Standardization week 22, 2023).

Demographic characteristics	Swabs (N=323), n (%)	
Age band (years)		
<1	11 (3.4)	
1‐4	43 (13.3)	
5‐14	27 (8.4)	
15‐24	24 (7.4)	
25‐44	89 (27.6)	
45‐64	77 (23.8)	
65‐74	27 (8.4)	
75‐84	18 (5.6)	
85+	7 (2.2)	
Sex		
Female	192 (59.4)	
Male	131 (40.6)	
Ethnicity		
White	150 (46.4)	
Asian	83 (25.7)	
Black	35 (10.8)	
Mixed	8 (2.5)	
Other	10 (3.1)	
Unknown	37 (11.5)	
Index of Multiple Deprivation quintile		
1 (most deprived)	42 (13)	
2	105 (32.5)	
3	52 (16.1)	
4	45 (13.6)	
5 (least deprived)	79 (24.6)	
Urban-rural classification		
City and Town	68 (21.1)	
Conurbation	198 (61.3)	
Rural	57 (17.7)	
Smoking status		
Active smoker	23 (7.4)	
Ex-smoker	50 (15.5)	
Nonsmoker	147 (46.4)	

### Swab Positivity

In total, swab positivity in the PIAMS study was 2.8% (9/323), with influenza A positivity at 0.3% (1/323), influenza B positivity at 1.6% (5/323), and RSV positivity at 0.9% (3/323). Multimedia Appendix 3 illustrates the proportion of swabs that were RSV and influenza positive in PIAMS practices compared with practices in the English National Sentinel Surveillance Network by week.

### Effects of POCT Results on Antimicrobial Prescribing 7 Days After POCT Testing

In total, there were 80 instances of antibiotic prescribing 7 days following POCT testing and no instances of antiviral prescribing 7 days following POCT testing in the PIAMS study. Table 3 illustrates the number of cases that prescribed antibiotics given the POCT result.

**Table 3.** Number of cases that prescribed antibiotics 7 days following point-of-care testing swab results in the point-of-care testing influenza on antimicrobial stewardship (PIAMS) study, a nested cohort study undertaken within the English National Sentinel Surveillance Network between January 20, 2023 (International Organization for Standardization week 3, 2023), and May 31, 2023 (International Organization for Standardization week 22, 2023).

POCT^a^ virology swab result		Antibiotic prescribed within 7 days of POCT virology swab result		Total
		Yes	No	
Positive, n		3	6	9
Negative, n		77	237	314
Total, n		80	243	323

a POCT: point-of-care testing.

The unadjusted OR for antibiotic prescribing given a positive POCT result was 1.54 (95% CI 0.38‐6.30; *P*=.55) compared with a negative POCT result. The adjusted OR for antibiotic prescribing, taking into account differences in age, sex, ethnicity, socioeconomic status as measured using the Index of Multiple Deprivation, urban-rural classification, and smoking status was 1.21 (95% CI 0.00‐1.78).

## Discussion

### Main Study Findings

A total of 10 general practices with a combined registered list size of 144,426 patients participated in this study. They integrated POCT into their clinical workflow, collecting 323 samples. Furthermore, 59.4% (192/323) of the samples were from female patients, and 39% (126/323) samples taken were from patients of Asian, Black, or Mixed ethnicity. The mean age of those swabbed was 37.28 (SD 25.05) years. In addition, 2.8% (9/323) of the swabs collected were positive, with influenza A positivity at 0.3% (1/323), influenza B positivity at 1.6% (5/323), and RSV positivity at 0.9% (3/323). Of the 9 POCT virology swab-positive cases, 33% (3/9) received antibiotics. Of the 314 POCT virology swab negative cases, 24.5% (77/314) received antibiotics. A statistically significant difference in antibiotic prescribing given a positive POCT result compared with a negative test was not found with an OR of 1.54 (95% CI 0.38‐6.30; *P*=.55).

### Implications of Our Findings

We have shown that in a postpandemic health service, POCT for respiratory viruses can be integrated into primary care workflows, although there was a wide variation in the rate of virological swabbing between practices. Our qualitative substudy identified 2 distinct POCT swabbing workflows—one led by clinicians and another managed by research nurses or health care assistants [27].

Key factors that influenced the adoption of each POCT swabbing workflow included the usability of the technology, the skill mix of primary care staff within the practice, the perceived ease of integration of POCT into routine clinical workflows, the availability of comprehensive staff training, the organizational readiness for change, and collective buy-in from all stakeholders [27].

The degree to which these different POCT swabbing workflow models were adopted could have accounted for the widespread differences in swabbing rates seen.

In addition, we have illustrated in Multimedia Appendix 3 that the number of swabs in the PIAMS practices and swab positivity found from POCT virology swabbing broadly reflected what was happening in the English national sentinel system over the same weeks [26]. Figure 2, from the English National Sentinel Surveillance Network, illustrated that RSV rates peaked in the early weeks of 2023, which was also seen in PIAMS practices (Multimedia Appendix 3), where RSV positive swabs were seen between ISO weeks 3 and 5, 2023.

However, our findings make it difficult to offer any recommendations on the impact of POCT on antimicrobial stewardship, as the number of patients prescribed antibiotics and antiviral medications following POCT virology swabbing results was low. Thus, our ORs for prescribing antibiotics given a positive POCT result compared with a negative POCT result were nonsignificant. OR for prescribing antivirals was not calculable as no antivirals were prescribed during the study. The absence of antiviral prescribing is of note despite the receipt of POCT-positive swab samples. This may be due to the only timing of the study at the end of the influenza season when there was a predominance of influenza B cases, which are much less likely to receive antiviral treatments despite evidence that these cases have comparable clinical outcomes to influenza A cases [28] and clinical guidelines recommending their use in POCT confirmed cases of influenza B [29].

### Comparison With Existing Literature

Our ORs for antibiotic prescribing given a positive POCT swabbing result of 1.54 (95% CI 0.38‐6.30; *P*=.55) contrasts with an earlier study conducted within the sentinel network before the pandemic in 2019, which found an OR of 0.4 (95% CI 0.2-0.8; *P*=.01) for antibiotic prescribing given a positive POCT result compared with a negative test, suggesting that antibiotic prescribing was less likely given a positive influenza POCT result compared with a negative result [11].

However, our results are consistent with a systematic review and meta-analysis of POCT in ambulatory care before the pandemic in 2019 which suggested that POCTs had no effect on antibiotic prescribing rates (relative risk 0.97, 95% CI 0.82-1.15; *I*^2^=70%) [30].

No further systematic reviews have been undertaken of POCT on influenza in the postpandemic primary care context, although research has suggested that overall antimicrobial prescribing for RTIs in the community reduced significantly by 12.4% during the pandemic winter season (December 202o to February 2021) compared with the prepandemic winter season (December 2019 to February 2020) [31-33], although there was a slight uptick in antimicrobial prescribing in 2022. Antimicrobial prescribing in primary care typically accounts for 80% (29/36.4) of total antibiotic prescribing in England [8], of which 46% (39.6/80.6) are prescribed for RTIs [34].

### Limitations of the Study

**Figure 3.**
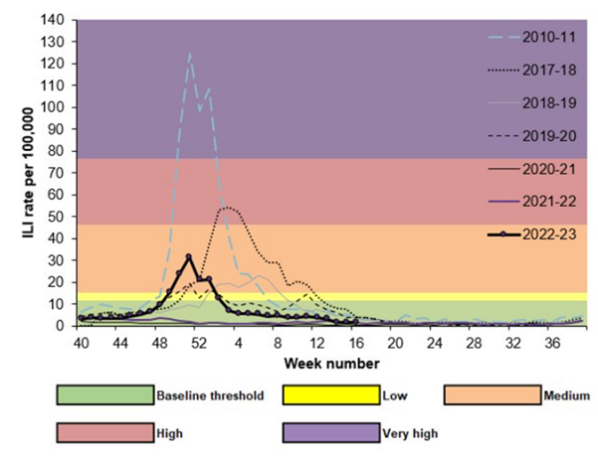
Weekly all-age GP influenza-like illnessv (ILI) rates in the English National Sentinel Surveillance Network for 2022‐2023 and past seasons [35].

The main weakness of our study was the small sample size as a result of the small number of practices included in the study and an earlier start to the seasonal influenza epidemic in October 2022 to May 2023 [35]. This was earlier than seen in previous years and is comparable with the peak of ILI seen in 2010‐2011 (Figure 3). A further weakness of our study was the lack of testing for respiratory viruses other than influenza and RSV. As illustrated in Figure 2, other respiratory viruses, such as COVID-19, were circulating in the community during our study. The lack of POCT analysis for microbes other than influenza and RSV, which could cause eligible symptoms and which may have had an impact on antimicrobial prescribing, could reduce the strength of our study to detect an effect of POCT on patient management in primary care. Some patients may also have had additional virological swabs sent to the reference laboratory for testing; however, this information was not available in this study and is unlikely to affect the prescribing of antimicrobials, given the significant delay between virological testing and the receipt of a result from the reference laboratory versus POCT.

### Conclusion

The practice of performing rapid testing for suspected viral illnesses had become an accepted norm for patients and clinicians alike during the COVID-19 pandemic [36]. As seasonal patterns of community spread of respiratory viruses are re-established following the pandemic, it is important to re-evaluate the impact of novel methods for rapid diagnosis and clinical or public health management of common respiratory viruses such as POCT. POCT tests might usefully be aligned with what the RSC sentinel network says is circulating.

Our study was performed immediately post pandemic and was disrupted by an earlier start to the influenza circulation in October 2022 to May 2023. Further research is needed to study the impact of POCT on clinical management in primary care, including its effects on antibiotic and antiviral stewardship and the cost-benefits of POCT in postpandemic UK general practice. This study illustrates the risk of having a narrow study window. Research teams planning studies of POCT testing associated with viruses that circulate seasonally should avoid narrow observation windows or risk low rates of identification of their target viruses.

## Supplementary material

10.2196/72322 Multimedia Appendix 1 Number of point-of-care testing swabs collected by week in the point-of-care testing influenza on antimicrobial stewardship (PIAMS) study, a nested cohort study undertaken within the English National Sentinel Surveillance Network between January 20, 2023 (International Organization for Standardization week 3, 2023), and May 31, 2023 (International Organization for Standardization week 22, 2023).

10.2196/72322 Multimedia Appendix 2 Swabbing rate per 1000 patients with eligible symptoms in point-of-care testing influenza on antimicrobial stewardship (PIAMS) study practices, a nested cohort study undertaken within the English National Sentinel Surveillance Network between January 20, 2023 (International Organization for Standardization week 3, 2023), and May 31, 2023 (International Organization for Standardization week 22, 2023). Eligible symptoms include patients registered within PIAMS study practice who were showing symptoms of influenza like illness (ILI), acute respiratory illness (ARI) or fever of higher than 37.5 °C.

10.2196/72322 Multimedia Appendix 3 Proportion of swabs that were respiratory syncytial virus and influenza positive in the point-of-care testing influenza on antimicrobial stewardship (PIAMS) study practices, a nested cohort study undertaken within the English National Sentinel Surveillance Network between January 20, 2023 (International Organization for Standardization week 3, 2023), and May 31, 2023 (International Organization for Standardization week 22, 2023), compared all practices with the English national sentinel network by week.

## Abbreviations

ARI: acute respiratory illness
GP: general practitioner
ILI: influenza-like illness
ISO: International Organization for Standardization
OR: odds ratio
PIAMS: point-of-care testing for influenza on antimicrobial stewardship
POCT: point-of-care testing
RCGP: Royal College of General Practitioners
RSC: Research and Surveillance Centre
RSV: respiratory syncytial virus
RTI: respiratory tract infection
