# Supplementary figures and images for: The Impact of Point-of-Care Testing for Influenza on Antimicrobial Stewardship in UK Primary Care: Nested Cohort Study
Source: JMIR Public Health Surveill. 2025 Jul 15;11:e72322. doi: 10.2196/72322 (PMC12283063; doi:10.2196/72322)

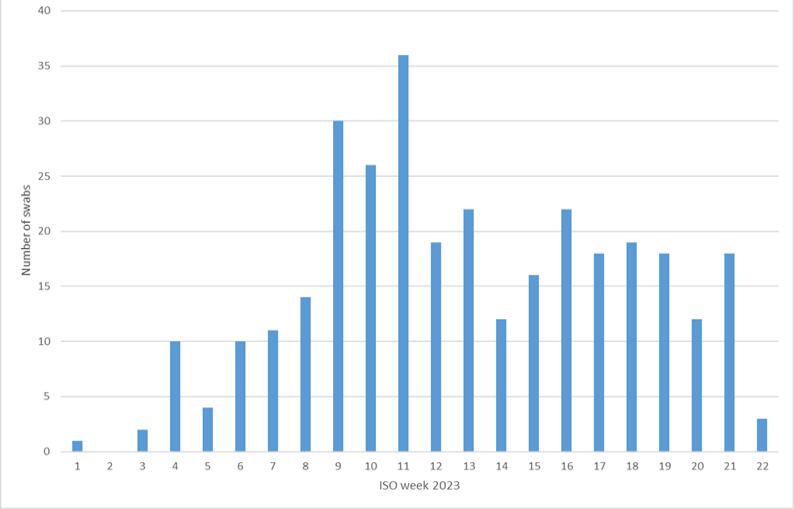

Supplement: Multimedia Appendix 1 [file publichealth-v11-e72322-s001.jpg]

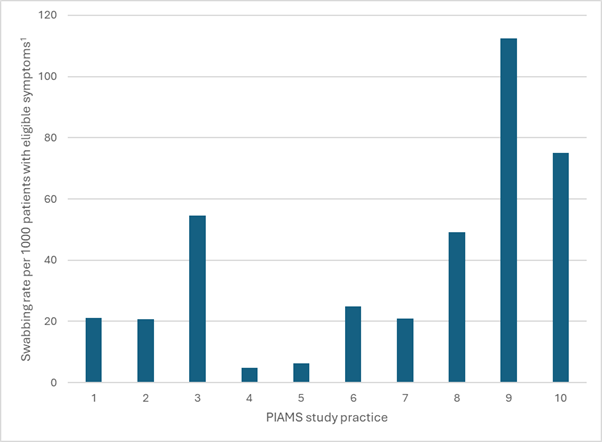

Supplement: Multimedia Appendix 2 [file publichealth-v11-e72322-s002.png]

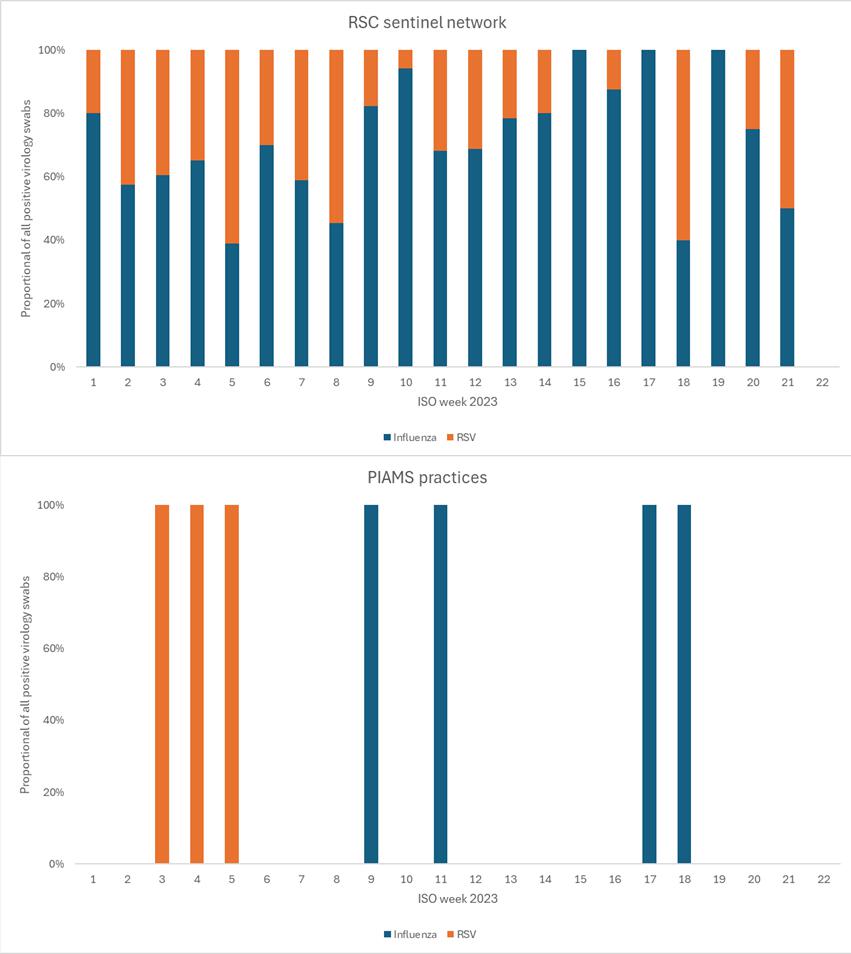

Supplement: Multimedia Appendix 3 [file publichealth-v11-e72322-s003.jpg]
